# A training algorithm for networks of high-variability reservoirs

**DOI:** 10.1038/s41598-020-71549-y

**Published:** 2020-09-02

**Authors:** Matthias Freiberger, Peter Bienstman, Joni Dambre

**Affiliations:** 1 IDLab-AIRO, Ghent University-imec, 9052 Ghent, Belgium; 2 Photonics Research Group, Ghent University-imec, 9052 Ghent, Belgium

**Keywords:** Electrical and electronic engineering, Photonic devices

## Abstract

Physical reservoir computing approaches have gained increased attention in recent years due to their potential for low-energy high-performance computing. Despite recent successes, there are bounds to what one can achieve simply by making physical reservoirs larger. Therefore, we argue that a switch from single-reservoir computing to multi-reservoir and even deep physical reservoir computing is desirable. Given that error backpropagation cannot be used directly to train a large class of multi-reservoir systems, we propose an alternative framework that combines the power of backpropagation with the speed and simplicity of classic training algorithms. In this work we report our findings on a conducted experiment to evaluate the general feasibility of our approach. We train a network of 3 Echo State Networks to perform the well-known NARMA-10 task, where we use intermediate targets derived through backpropagation. Our results indicate that our proposed method is well-suited to train multi-reservoir systems in an efficient way.

## Introduction

In recent years, brain-inspired approaches to computing have gained increased attention in the scientific community due to their potential for low-energy, high-performance computing. Reservoir computing is one of these approaches, and exploits the rich dynamics of nonlinear systems in order to perform computations. Originally proposed as a method to train recurrent neural networks (RNNs), due to its universal applicability on all kinds of nonlinear dynamical systems beyond RNNs, it has experienced wide popularity in the unconventional computing community.

The essential idea of reservoir computing is that, instead of training all weights of an RNN, one can simply train the output layer of an RNN using linear regression, in order to combine the natural hidden states of the RNN to a desired signal. The approach exploits the fact that a fixed-weight RNN constitutes a nonlinear dynamical system, which transforms the input data into a high-dimensional space. In this high-dimensional space, linear regression is sufficient to find a suitable combination of necessary signal components of the input signal and combine them to a desired solution^1^. Since a wide range of nonlinear dynamical systems can be performed reservoir computing with, it stands to reason to also apply these ideas to real physical systems. In recent years, reservoir computing with physical systems has successfully been demonstrated, among others, for mechanical^2,3^, memristive^4–6^, spintronic^7,8^ biological^9^ as well as photonic reservoirs^10–13^. The most common varieties of linear regression used here are ordinary least squares (OLS) for offline learning^2,3,7,8,10–12^ as well as least mean squares (LMS) or recursive least squares (RLS) for online learning^9,14,15^. Depending on given hardware constraints, systems are also trained using reinforcement-learning-like or blackbox approaches^16–18^. A comprehensive review on recent advances on physical reservoir computing is given in^19^.

A particular intriguing property of a large class of physical reservoirs is the fact that the intrinsic parameters determining their performed computation, that is, the coefficients of their system matrices, do not need be controlled exactly but can be worked with as dictated by physics. As long as the determining parameters follow a certain probability distribution necessary to solve a given task, variations between fabricated physical reservoirs can be embraced, which favors simple and cheap manufacturing processes. An example for such a type of reservoirs are integrated photonic reservoirs^12^, where every silicon-on-insulator fabricated reservoir is unique in how it computes due to varying optical waveguide properties.

Beyond fabrication variabilities, another factor contributing to the inherent stochasticity of physical reservoirs is the presence of naturally occurring noise. Contrary to manufacturing variabilities which are fixed at design time, noise varies persistently. Machine learning literature^20^ suggests that noise can have a beneficial impact on the training process, depending on its distribution. The general interactions, benefits and challenges of naturally occurring noise on training and evaluating physical reservoirs are worthwhile subjects to be researched in future work. Within this work though, we focus on the former source of stochasticity, i.e. variabilities introduced at design time, through the fabrication process.

Despite recent successes in the field of physical reservoir computing, it is well known in the reservoir computing community that there are bounds to what one can achieve simply by making a reservoir larger and using more state signals in the readout. This is mostly due to the fact that the states are usually highly correlated due to the interactions inside the reservoir. This makes it difficult to exploit the residual information that is added by additional states as the state space grows larger. In physical reservoirs, noise and measurement inaccuracies effectively constrain the exploitation of state information, performance as a function of reservoir size tends to saturate fast. In addition, for physical reservoirs manufactured in integrated circuits (e.g.^12^), fabricating larger reservoirs increases the technological challenge, e.g., due to routing problems and losses, as well as cost and yield-related constraints on the chip size. In summary, in order to move to more complex tasks, simply making reservoirs larger is not enough.

**Figure 1:**
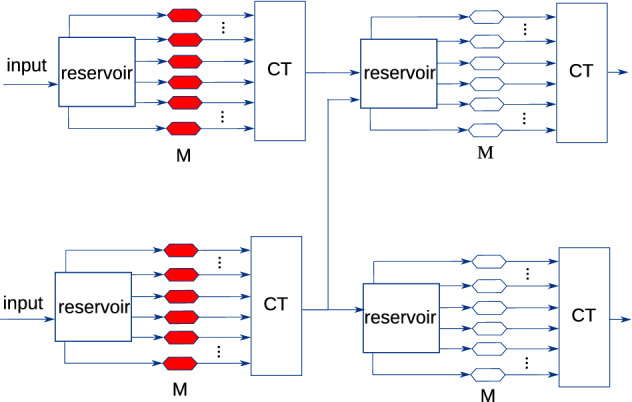
An example of a custom multi-reservoir architecture consisting of several physical reservoirs. Modulators (weighting elements) highlighted in red cannot be trained by traditional reservoir computing methods, due to a missing target signal. *M* modulator, *CT* combiner tree.

Therefore, in analogy to deep learning^21–23^, which achieves excellent performance through many subsequent nonlinear transformations of the input data, a paradigm of deep physical reservoir computing appears to be promising, where we optimise custom multi-reservoir architectures of physical reservoirs such as the one illustrated in Fig. 1.

It is not clear though, how such a network of connected physical reservoirs can be trained. While single reservoirs are usually trained using linear regression, this is not possible for all reservoirs in a multi-reservoir network: a desired signal, which is necessary to train each reservoir, is only available for the final reservoir(s), that generate(s) the output (see Fig. 1 for illustration). Accordingly, recent approaches to physical multi-reservoir architectures^24–26^ usually constrain themselves to architectures where only the final output labels need to be known in order to train the setup using linear regression. This unfortunately implies that many possible architectures which might deliver good performance need to remain unexplored.

Error backpropagation on the other hand, could in principle be used to train multi-reservoir architectures such as the one shown in Fig. 1. For many physical reservoir implementations though, the state update matrix of the corresponding dynamical system varies naturally, and can not easily be known or controlled. This rules out backpropagation as a training algorithm for these devices.

Since this problem occurs for a variety of different substrates, within this work, we introduce an abstract model to study the problem at hand in closer detail. We seek to train networks of high-variability reservoirs, which are sampled from a probability distribution. We assume all reservoir networks to be of a given, predetermined architecture. The input and connection weights of individual reservoirs in a sampled network on the other hand, vary according to predefined distributions. We propose an approach to derive intermediate targets for such networks through backpropagation. These intermediate targets can then be used to train networks sampled from the corresponding distribution using linear regression. We evaluate this framework on the NARMA-10 task using a predefined multi-reservoir architecture composed of Echo State Networks (ESNs). For comparison, we employ a baseline approach which we have derived analytically for this task on the same architecture, as well as a classically trained single ESN of identical size. We discover that backpropagation autonomously learns the same intermediate targets which we have found by inspecting the system, and assigning desired signals analytically. We consider this as a proof-of-concept for our investigated training approach: we show that backpropagation in simulation is suitable to find targets for physical multi-reservoir architectures which can then be trained in a classical way. Furthermore, we find that our proposed approach slightly outperforms training a single reservoir of the same size. In view of the fact that in literature the NARMA-10 task is considered to be largely solved, this indicates that the performance gap between single-ESN and multi-ESN systems for different, unsolved tasks to be larger, provided that a suitable multi-reservoir architecture can be found.

## Results

### Necessary ingredients for deep physical reservoir computing

Upon closer inspection, several ingredients that have led to the success of deep learning can be identified:

- Suitable tasks: The rise of enormous datasets of several hundreds of gigabytes has been a major driver of deep learning, as millions of parameters need to be trained in a deep neural network.
- Suitable architectures: Architectures of deep neural networks are mainly found through experimentation. Specialised deep learning frameworks allow for very rapid prototyping of new architectures through fully automatic gradient computation and intrinsic parallelisation of matrix multiplications on widely available high-performance consumer graphics processing units (GPUs). Furthermore, new activation functions, as well as optimization and regularization techniques have made the training of deep networks possible.
- Suitable training algorithms: error backpropagation^27–32^, or short, backpropagation, a powerful, yet simple algorithm permits the optimization of any known, parameterised, (semi-)differentiable function, which deep neural networks are.

If we now consider these ingredients with respect to a potential paradigm of deep physical reservoir computing, we find that suitable tasks are likely to be found for any operations on low-dimensional time-series data. Suitable architectures on the other hand will most likely have to be found in simulation, empirically, or possibly through search strategies^33,34^, as they are in deep learning. Deep learning frameworks^35,36^ permit the construction of fully differentiable physics simulators^37,38^, which present themselves as a suitable tool for multi-reservoir architecture exploration.

We encounter a challenge, though, when considering the training algorithm to be used. The output layers of physical reservoirs are usually trained using various kinds of linear regression^39^, which typically only require the desired output signal as well as the knowledge of the hidden layer outputs. In order to train structures such as the one shown in Fig. 1, we would also need to know desired signals for all intermediate outputs in order to train the reservoirs which generate them.

Using backpropagation instead of linear regression to train the readouts in a network of physical reservoirs in simulation may appear as an obvious solution. Yet, this proves to be infeasible for a large class of physical reservoirs mentioned above, whose parameters can not be controlled precisely due to natural variations. As backpropagation requires full observability of all reservoir parameters, which differ for every single reservoir, and obtaining them through measurement is usually infeasible, these systems can not be trained using standard backpropagation. With prospects for future deep architectures of physical reservoirs, we argue that finding a way to train complicated architectures of these reservoirs in an efficient fashion is desirable.

### Related work

Gallicchio et al.^40^ propose to scale reservoir computing by stacking a large amount of smaller reservoirs, where each reservoir injects its state vector as input into the next reservoir. The states of all reservoirs are then fed into one overall readout. A similar concept has been implemented for delayed-feedback reservoirs by Penkovsky et al.^24^, except that here reservoirs are connected in a bidirectional way, using both forward and backward connections, in addition to being connected to a global readout. While both approaches perform well, they are challenging to implement with physical reservoirs integrated on a chip due to their high wiring effort, which subjects them to almost the same routing issues and constraints as for large monolithic reservoirs.

Approaches based on standard ensembling techniques from machine learning, which are cheaper in terms of design constraints and wiring effort, have been evaluated for integrated photonic reservoirs in^26^. Yet, the introduced approaches are rather general and do not take into account, that better performance might be achieved by a network of reservoir modules which has been tailored specifically to a given task.

Similar work has also been done by Keuninckx^25^, who found that stacking electronic delay-line reservoirs improves performance on signal equalisation tasks. All the approaches above have in common, that they usually constrain themselves to architectures where only the final output labels need to be known in order to train the setup using linear regression. This unfortunately implies that many possible architectures which might deliver good performance need to remain unexplored.

Another related line of research, though it can not be considered reservoir computing, is the construction and training of feed-forward neural networks in hardware. In terms of optical systems, a notable example is the work of Shen et al.^41^, who demonstrate a electro-optical feed-forward neural network, where all occurring matrix multiplications are performed fully optical. Since these multiplications are performed by means of a programmable mesh of Mach–Zehnder-interferometers^42^, the system can be pretrained, and is not trained in-place such as the systems we consider ourselves with. Hughes et al.^43^ on the other hand propose an in-situ backpropagation training algorithm for integrated photonic feed-forward neural networks based on the adjoint variable method^44,45^. This approach only requires observability of the neuron outputs rather than its weights. It seems promising to investigate an extension of this algorithm to backpropagate through physical reservoirs. Yet, one must keep in mind that the algorithm in^43^ uses stationary signals to perform backpropagation on feed-forward neural networks. The step to using transient, often complex-valued signals, in order to perform backpropagation through time does not appear straight-forward. Furthermore, this algorithm requires full observability of all signals at the corresponding neurons, which is not necessarily given per se for integrated physical reservoirs^18^.

### Proposed approach to train networks of reservoir architectures

In this work we choose a different path and abstract the problem as follows: training custom multi-reservoir networks of physical reservoirs implies that one needs to train networks of high-variability reservoirs without direct use of backpropagation. In more detail, we seek to find a general way to train multi-reservoir networks sampled from a certain probability distribution. While the network architecture remains the same for all sampled instances, we assume that input and connection weights of the reservoirs differ for every instance. In such a problem setting, we propose to use backpropagation to derive intermediate target signals. These intermediate target signals can then be used for all sampled networks of a given architecture, rather than training each sampled network instance with backpropagation. One therefore derives target signals by training a sampled multi-reservoir network using backpropagation. Thereafter, the intermediate signals between reservoirs, which have been found by backpropagation for that sampled network, are used as target signals to train future sampled networks.

**Figure 2:**
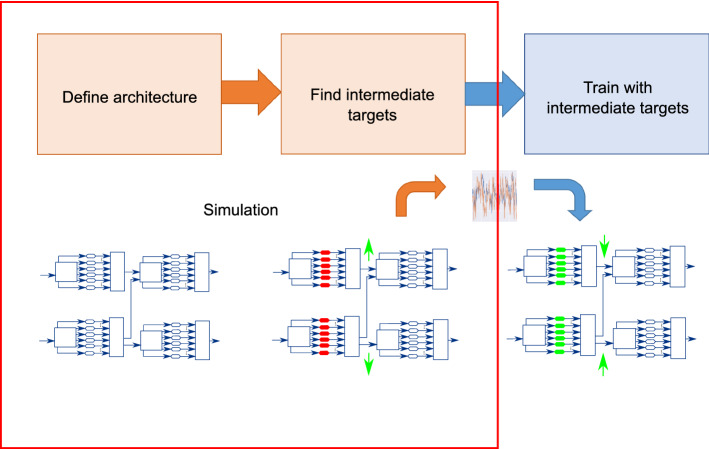
Proposed approach to train custom high-variability networks of reservoirs to be applied to physical reservoirs with intrinsic variability. In simulation, a suitable architecture is defined for a given task, after which intermediate target signals are derived for that architecture. These target signals are then used to train the real physical devices using classical reservoir training algorithms. Steps within the red frame are still to be performed in simulation, steps outside are performed on the actual hardware.

The block diagram shown in Fig. 2 gives an overview of the approach. The overall approach consists of 3 steps. One first defines a suitable architecture. Once such architecture has been found, intermediate targets can be derived for that specific architecture, after which these targets are used to train all devices in the reservoir network using linear regression. Transferring our abstracted approach to actual physical hardware would of course imply that both the architecture definition step as well as the derivation of the targets needs to be conducted in simulation, as indicated by the red frame in Fig. 2: a simulated network of physical reservoirs is trained using backpropagation. Thereafter, the found intermediate signals between simulated reservoirs, which have been found by backpropagation, can be used as target signals, to train an identical architecture of real physical devices using classical training approaches.

Within this work we assume a certain network architecture to be given, and primarily concern ourselves with training networks of high-variability reservoirs for this architecture. To investigate the feasibility of training networks of sampled reservoir systems as proposed, we use Echo State Networks (ESNs) as example systems. ESNs are well understood and known to work well for a number of tasks. They are therefore suitable surrogates for the wide variety of physical reservoirs, and ensure independence from specific hardware implementations. This implies that *all reservoirs in this work have been implemented in software*. The very specific challenges of transferring this approach to real physical reservoirs, which might arguably occur, will be addressed in future work. Dependent on reservoir type and substrate used (e.g. photonic, spintronic, memristive, mechanical), different challenges might occur. Therefore the transfer of this approach to physical reservoirs should be addressed by experts on the corresponding substrate.

### Used benchmark task

**Figure 3:**
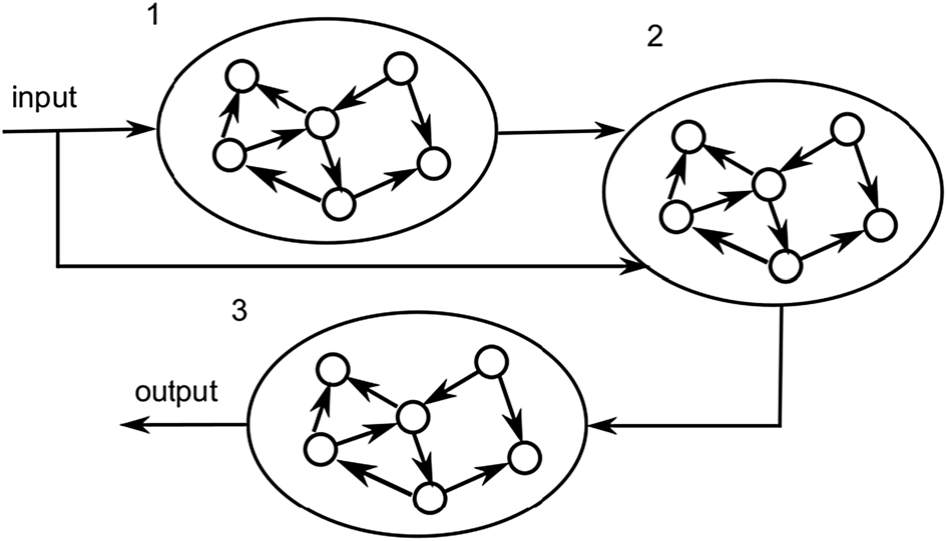
System architecture of three connected Echo State Networks for which intermediate desired signals have been derived using backpropagation.

We used the Nonlinear Autoregressive Moving Average system identification task of order 10 (NARMA-10)^46^ as a benchmark for our new training approach. The NARMA-10 task is particularly useful to us, since, contrary to many other tasks, it can easily be decomposed in an analytical way, and be mapped to a multi-reservoir architecture such as the one shown in Fig. 3. We refer to the target signals derived through such an analytical decomposition as *analytical targets* and to the trained system using these targets as the *analytical baseline*. These analytical targets can be compared against more general target derivation approaches, which are useful for different tasks, where such analytical decompositions might be much harder to find.

We evaluate our proposed approach on the NARMA-10 task using the architecture shown in Fig. 3, and compare it to the analytical baseline on the same architecture as well as a classic monolithic reservoir. For both the analytical baseline and our proposed approach individually, we sample from the weight distribution of the architecture shown in Fig. 3. For the classic monolithic reservoir, a similar sampling procedure is applied. For all experiments, we use the normalised mean squared error (NMSE) as an error measure:

1 $$\begin{aligned} e_{\text {NMSE}} = \frac{1}{N} \sum _{n=1}^{N} \frac{(\hat{y}(n) - y(n))^2}{\sigma _y^2}, \end{aligned}$$

where $\hat{y}(n)$ is the prediction of the trained network, and $\sigma _y^2$ is the variance of the desired signal *y*(*n*). In order to account for the variability of our reservoir networks, we compute the average obtained NMSE on all sampled networks per training approach. As already mentioned above, within this work, we use networks of ESNs as abstractions of a broad class of physical reservoirs.

### Performance achieved by investigated approaches

We started out by training 10 monolithic ESNs of 300 nodes each, as well as 10 multi-ESN systems with analytically derived targets as explained above. Each multi-ESN system consists of three 100-node ESNs. Table 1 shows the results of these experiments in rows 1 and 2.

**Table 1:** Experiment results. Single ESN and analytical targets rows denote mean and standard deviation of NMSE test error on the NARMA-10 task of single-ESN and multi-ESN baselines respectively. Learned targets rows denote NMSE test error on the NARMA-10 task of models trained to derive targets in the left column, as well as the means and standard deviation of NMSE test errors achieved when training new reservoirs with the respective derived desired signals in the right column. Mean and standard deviation computed over 10 reservoir networks with randomly initialised weights.

	NMSE backprop.	NMSE linear reg. (10 ESN)
Single ESN	–	0.035 ± (0.016)
Analytical targets	–	0.020 ± (0.013)
Learned targets (Model 1)	0.080	0.072 ± (0.017)
Learned targets (Model 2)	0.039	0.031 ± (0.003)
Learned targets (Model 3)	0.058	0.046 ± (0.006)
Learned targets (Model 4)	0.106	0.064 ± (0.008)
Learned targets (Model 5)	0.064	0.051 ± (0.004)

Our monolithic ESN system achieves an average NMSE of 0.035 with a standard deviation of 0.016 on the test set, which is close to the results reported in^47^. Note that, while the ESNs in^47^ are smaller, their reservoir state matrix is concatenated with a squared version of itself prior to training/evaluation in order to enhance state richness and utilise an additional output nonlinearity. We omit these steps in favor of simplicity and to ensure that our results allow us to judge the feasibility of our approach for future hardware implementations, where such operations would need to be omitted or implemented by a circuit.

As intended, the engineered multi-ESN system outperforms the monolithic ESN baseline with an average test NMSE of 0.020 at a standard deviation of 0.013. This also delivers proof-of-concept that multi-ESN architectures with a suitable task distribution among reservoirs can outperform a single reservoir with an identical total number of nodes.

After establishing the quality of our analytical task decomposition, we derived 5 sets of intermediate targets using our proposed method: we trained 5 new multi-reservoir systems with the same architecture and ESN hyperparameters, using backpropagation. The center column of Table 1 shows the achieved NMSE of the systems trained to derive learned targets, which we refer to as Model 1 to Model 5. We find that the performance of these models varies a lot, mainly due to large variations in the convergence of the backpropagation algorithm. The model achieving the best NMSE of 0.039, namely Model 2, is still outperformed by both the monolithic ESN as well as the multi-ESN-system with analytical targets.

Nevertheless, we extracted the intermediate signals of each trained network in response to the train, validation and test data. In the next step, we used these signals as intermediate target signals for train, validation and test set respectively for 10 new multi-ESN systems for each of the 5 networks trained. As a reminder, these 10 new random systems symbolise different instantiations of physical reservoirs, for which we do not have access to the corresponding state update matrix of the system. For the targets derived from Model 1 to Model 5, this resulted in the average NMSE scores as shown in the right column of Table 1.

It turns out that the networks trained with transferred, learned targets, mostly outperform the corresponding target-signal-giving model itself. This observation may be explained with the fact that the former are trained with ordinary-least-squares (OLS) linear regression. Contrary to stochastic gradient descent with backpropagation, OLS locates the global minimum of the loss function. The networks generating the learned target signals on the other hand, are trained with backpropagation, which usually locates slightly less well-performing local minima. Therefore, the latter might exhibit, slightly weaker, more varying performance.

This effect also implies that multi-reservoir architectures trained with targets from Model 2 slightly outperform the monolithic ESN on average. The rather low standard deviation of 0.003 on the test loss also indicates that the derived desired signals work for a wider range of ESNs with random weights, at least when they are derived from a model where backpropagation has converged to a sufficiently good local minimum. Nevertheless, one needs to consider here that the NARMA-10 task, despite its suitability as a benchmark task, can be considered largely solved for ESNs. Therefore we would like to emphasise, that the fact that our proposed method indeed works and can be used to find intermediate targets for suitable architectures, is the more important finding here. Given a suitable architecture, our method could also be useful to outperform monolithic reservoir systems on harder tasks, where targets can not be found in an analytical way as it is possible for NARMA-10.

## Discussion

**Figure 4:**
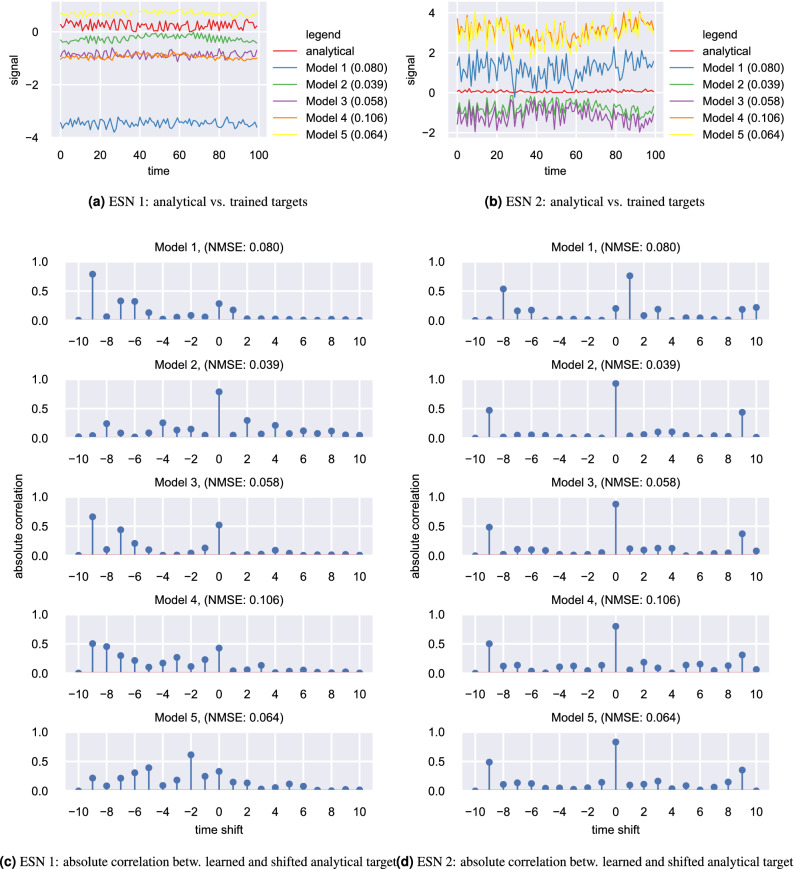
(**a**,**b**) Show the trained target signals of the 5 target-generating models for the first and second intermediate ESN (named ESN 1 and ESN 2) respectively. (**c**,**d**) Show the absolute correlation between trained target signals and shifted analytical target signals as a function of timeshift, again for the first and second intermediate ESN respectively.

We compared the trained intermediate targets of the individual reservoirs in our architecture to the analytical targets we derived by hand. Figure 4a shows the trained targets for the first intermediate ESN (ESN 1 in Fig. 3), in comparison to the analytical targets for the first 5 target-generating models. Upon visual inspection, the trained targets of Model 2, which achieved best performance among the models that provided the desired signals, look quite similar to the analytical targets. Model 3 and Model 5, who performed intermediately, appear to have some resemblance to the analytical targets as well, while models 1 and 4, the worst performing models, show only very remote resemblance to the analytical targets.

If we consider the analytical and trained targets for the second ESN (ESN 2) in our trained architecture, as shown in Fig. 4b, we observe a very similar behavior. Here, all trained targets appear to be either correlated or anti-correlated with each other as well as with the analytical desired signal. This desired signal has a much smaller amplitude, though.

To confirm these observation quantitatively, we plot the absolute correlation between the trained targets and the time-shifted analytical targets as a function of time shift in Fig. 4c,d for ESN 1 and ESN 2 respectively. In Fig. 4c, we can see that the targets of Model 2 show a strong correlation with the analytical targets without any timeshift. Model 3 on the other hand shows some correlation with the analytical targets without any timeshift, but shows even larger correlations with the analytical target shifted by 9 time steps to the right, i.e. a target signal whose every value occurs 9 timesteps earlier. Since the analytical target signal is meant to train a reservoir to act as a delay line, which delays the signal for 9 timesteps, this shifted analytical signal corresponds to the original input signal.

That implies that the first reservoir of Model 3 (ESN 1), got partly trained by backpropagation to act as a delay line, but also still leaks some of its original input signal. This leakage effect seems even worse for Model 1, where the first ESN basically copies the input and the delay line task has to be performed by subsequent reservoirs. Model 5 appears to have found some kind of middle ground and implemented a delay line of 7 timesteps. This suggests that the model utilises ESN 1 as a delay line, but does not fully delay for the requested number of steps, but leaves the remaining delay steps to be implemented by subsequent reservoirs. Finally, Model 4 appears to exhibit a superposition of all behaviors discussed above: its output signal appears to be a mixture of several input signals with various levels of delay.

Moving on to the absolute correlation between the trained targets and the shifted analytical target signal in ESN 2, we can see from the corresponding needle plot in Fig. 4d, that the correlation between the analytical target and the trained targets is often even stronger here. Trained targets from models 2 to 5 show a very high absolute correlation with the unshifted analytical target signal. Model 1 forms a slight exception here, as it rather shows a high absolute correlation with the target signal shifted by one timestep to the right, which corresponds to a delayed analytical target signal. Judging from the comparably weak performance of Model 2, this delay seems to affect the ability of the subsequent ESN 3 to correctly form the final result.

From the above observations we conclude that backpropagation seems to learn targets which are highly correlated with the analytical targets the system has been designed for. Furthermore, the performance of the model trained with backpropagation (and therefore also the performance of the systems using the derived target signals) is highly correlated with how well backpropagation manages to learn targets similar to the original analytical targets of the architecture.

In conclusion, we have proposed a new training method that can in principle be used to train any given multi-reservoir network of physical reservoirs that do not permit direct training through backpropagation. Instead, the method uses backpropagation to derive desired signals for training the individual reservoirs in the network. We established that a specialised engineered multi-ESN architecture can outperform a general-purpose single reservoir system for the chosen task, and have shown that it is possible to derive similar targets from scratch by using backpropagation on the same architecture. While this training method outperforms the classical approach on average only by a small margin, it can be of use to overcome technological constraints which prevent the manufacturing of larger reservoirs to scale up performance. Furthermore, for different, unsolved tasks, this method may bring more significant improvements.

Future work will be directed at the critical points of this approach. First, convergence and obtained results of our approach can still be optimised by refining the backpropagation process through multiple layers of reservoirs. Second, a workflow needs to be established, which specifies how to find suitable multi-reservoir architectures for tasks, where the architecture can not be derived from the task itself. Finally, the approach needs to be applied to actual physical reservoirs, and any arising challenges intrinsic to this step need to be addressed.

This work focused on finding a training approach when the architecture itself has already been designed. Finding the right architecture is a different problem, that could be addressed with technically similar approaches as found in deep learning: by trial and error or by using some the recently developed architecture search strategies^33,34^. In order to be able to apply this work to actual physical reservoirs, such as integrated photonic reservoirs, a differential simulator for the given reservoir technology needs to be available. For integrated photonic reservoirs, one could use the recently released PhotonTorch framework^37^, which uses the automatic differentiation engine of Pytorch^36^ to conduct backpropagation through photonic reservoirs in simulation. Previous work^13,48^ indicates that this is in principle feasible. Since the hyperparameters of integrated photonic reservoirs are not directly tuneable, one would need to control the reservoir’s tradeoff between nonlinearity and memory in another way though. For instance, one could generate the required dynamics by adding nonlinear elements in strategic positions in the reservoir. Possible candidates for such elements in optical reservoirs are silicon optical amplifiers^49^ or ring resonators^50^. These components also have parameters (e.g. bias current, resonance wavelength) which can further be optimised in a similar way as we did with the hyperparameters of the ESNs in this work. This optimisation process would have to happen in simulation, likely while finding a suitable multi-reservoir architecture.

## Methods

### Analytical task decomposition

The Nonlinear Autoregressive Moving Average system identification task of order 10 (NARMA-10)^46^ is defined as follows:

2 $$\begin{aligned} y(n+1) = 0.05 y(n) \sum _{k=0}^9 y(n-k) + 0.3 y(n) + 1.5 u(n - 9) u(n) + 0.1, \end{aligned}$$

where *y*(*n*) denotes the desired output signal and *u*(*n*) denotes a random input sequence uniformly distributed in [0, 0.5]. For $t < 0$, *u*(*n*) and *y*(*n*) are by definition 0.

We decomposed this task into three subtasks to be mapped on the reservoirs in the multi-reservoir network of Fig. 3. As there are delayed input components in the task, a first subtask (Module 1) is a delay line with the desired signal

3 $$\begin{aligned} y^{(1)}(n) = u(n - 9). \end{aligned}$$

Module 2 multiplies the delayed version of the input signal with its original version:

4 $$\begin{aligned} y^{(2)}(n) = u(n) \hat{y}^{(1)}(n), \end{aligned}$$

where $\hat{y_{1}}(n)$ is the output of module 1, which only approximates its desired output. Finally, module 3 converts the output of module 2 into the final NARMA-10 output:

5 $$\begin{aligned} \begin{aligned} y^{(3)}(n+1) = 0.05 y^{(3)}(n) \sum _{k=0}^{9} y^{(3)}(n-k) + 0.3 y^{(3)}(n) + 1.5 \hat{y}^{(2)}(n) + 0.1, \end{aligned} \end{aligned}$$

where $\hat{y}^{(2)}(n)$ is the output of module 2. If the approximations made by the three modules are perfect, this decomposition exactly reproduces the original task.

### Architectures

As modules in the multi-reservoir architecture of Fig. 3, we used ESNs of 100 nodes. Because the aim was to use backpropagation on the architecture, we used Exponential Linear Units (ELUs)^51^ as nonlinearities to ensure a sufficient propagation of gradients through the network.

To verify whether our engineered solution is competitive, we compared it to a monolithic reservoir with similar resources, i.e., an ESN with 300 nodes. While the main point of this work is not to outperform previous training approaches and networks on NARMA-10, but rather finding an efficient way to train multi-reservoir systems, we consider establishing a simple baseline as a point of reference mandatory. We used a hyperbolic tangent nonlinearity for the single-ESN system since this nonlinearity exhibited the best performance for single-reservoir systems in preliminary experiments.

We trained our multi-reservoir system in three different ways. First, we evaluated the engineered task decomposition by training the three ESNs in the system to fit the three desired signals respectively. The ESNs were trained incrementally: each ESN takes input from previously trained ESNs and/or the input signal to solve its assigned subtask. This way, each ESN is trained to be maximally robust to approximation inaccuracies of its predecessor(s).

Second, after having shown that the NARMA-10 task can be solved by decomposing it by hand and mapping it to a suitable reservoir architecture, we trained the multi-reservoir architecture from the previous step as a whole (using different, randomly initialised ESNs) using backpropagation to automatically find good target signals.

Third, we evaluate the transferability of the trained target signals. Here, the best performing network obtained in the previous step was used to generate target signals for networks of different, randomly initialised ESNs from the same class.

### Training and parameter tuning

We train and evaluate all ESN networks using PyTorch^36^. To train our networks, we generated 3 sequences of 100,000 samples each for train, validation and test sets by sampling from a random uniform distribution on the interval [0,0.5]. The regularization strength of our ridge regression reservoir readouts was determined by performing grid search in combination with 5-fold cross-validation on the train set. For both the monolithic ESN as well as the multi-reservoir architecture with analytical targets, the validation set was used to find the hyperparameters that are intrinsic to the reservoir: spectral radius, input scaling, and bias scaling, leak rate, as well as the sparsity of the reservoir input and connection matrices. All ESN hyperparameters were tuned using hyperopt^52^. More information on the tuning process as well as final hyperparameter settings for each ESN can be found in the supplementary material.

The found reservoir hyperparameter values from the previous step were also used in the networks trained to learn intermediate signals. We have trained our networks using backpropagation through time with a batch size of 60 samples for 120 epochs. We used Adam^53^, starting with a learning rate of 0.0005. After 60 epochs we divided the learning rate by 2. We applied a weight decay $\lambda = 0.0001$. We have used standard deep learning techniques to improve training convergence for our networks: We initialised the reservoir readout weights to be orthogonal^54^, used gradient clipping^55^ as well as a technique similar to batch normalization^56^. For the input signal of every reservoir, we trained a common amplification factor, as well as a common bias of the input signal, prior to injecting it to the input weight matrix. Finally, we applied a simple form of early stopping: Every 10 epochs in the training process, we recorded a snapshot of the current weights used in the network as well as the validation score it obtained. After 120 epochs, we chose the weight set with the lowest validation score. Mind that we took care to solely use techniques which likely can be applied in simulated networks of integrated physical reservoirs as well. More information on the whole training process can be found in the supplementary material.

## Supplementary information

Supplementary Information.

## Data Availability

An equivalent data set can be obtained by generating random input sequences uniformly distributed in [0, 0.5]. The corresponding labels are computed according to Eq. (2).
