# Supplementary material for: A training algorithm for networks of high-variability reservoirs
Source: Sci Rep. 2020 Sep 2;10:14451. doi: 10.1038/s41598-020-71549-y (PMC7468150; doi:10.1038/s41598-020-71549-y)
Supplement: Supplementary file 1 — Supplementary Information. [file 41598_2020_71549_MOESM1_ESM.pdf]

Supplementary Material: A training algorithm for networks of high-variability reservoirs

Matthias Freiberger, Peter Bienstman and Joni Dambre

1 Details on the used training algorithm

1.1 Used models and architecture

As already introduced in the main text, we use Echo State Networks (ESNs) to evaluate our proposed training algorithm, defined by the equations

$$\mathbf{x}(n+1) = (1-a)\cdot\mathbf{x}(n) + a\cdot f(\mathbf{W}_{\text{res}}\mathbf{x}(n) + \mathbf{b}_{\text{res}} + \mathbf{W}_{\text{in}}\mathbf{v}(n)), \tag{1}$$

where n is the timestep, $\mathbf{x}(n)$ is the state vector of the ESNs nodes, $\mathbf{v}(n)$ is a vector holding the input to each reservoir node at timestep n and $\mathbf{W}_{\text{in}}$ and $\mathbf{W}_{\text{res}}$ are the matrices holding the fixed weights of the input and hidden layers respectively. $\mathbf{b}_{\text{res}}$ is fixed bias vector. $f(\mathbf{h})$ is a nonlinear function which is computed elementwise on the hidden layer activation vector $\mathbf{h} = \mathbf{W}_{\text{res}}\mathbf{x}(n) + \mathbf{W}_{\text{in}}\mathbf{v}(\mathbf{n})$. a is the leaking decay rate, which determines the response of the system to various input timescales. The output layer of the reservoir is defined on top of the reservoir state vector $\mathbf{x}(n)$ as

$$\hat{y}(n) = \mathbf{w}_{\text{out}}\mathbf{x}(n) + b_{\text{out}}, \tag{2}$$

where $\mathbf{w}_{\text{out}}$ is the trainable readout weight vector and b_{out} is a trainable bias term.

For all conducted experiments, we initialise $\mathbf{W}_{\text{in}}$, $\mathbf{W}_{\text{res}}$ and $\mathbf{b}_{\text{res}}$ sampling from a uniform distribution with range $[-0.5, 0.5]$, after which the matrices are scaled and sparsed out as dictated by the corresponding reservoir hyperparameters: spectral radius, input scaling, bias scaling, as well as input and connection sparsity.

1.2 Training ESNs with linear regression

Classically, ESNs are trained using L2-regularised linear regression [1], where we find $\mathbf{w}_{\text{out}}$ such that $\hat{y}(n)$ approximates the desired signal $y(n)$. We do so by collecting a time-trace of the ESN state vector $\mathbf{x}(n)$ as we present an input signal to the reservoir and arrange our collected state vectors in the rows of a reservoir state matrix $\mathbf{X}$

$$\mathbf{X} = \begin{pmatrix} \mathbf{x}(n_0)^\intercal, 1 \\ \mathbf{x}(n_0+1)^\intercal, 1 \\ \mathbf{x}(n_0+2)^\intercal, 1 \\ \vdots \\ \mathbf{x}(n_1)^\intercal, 1 \end{pmatrix}. \tag{3}$$

Note the incorporation of a column vector of ones in the rightmost column of $\mathbf{X}$. This allows us the find $\mathbf{w}_{\text{out}}$ and b_{out} jointly by means of the L2-regularised Moore-Penrose pseudoinverse as

$$\begin{pmatrix} \mathbf{w}_{out} \\ b_{\text{out}} \end{pmatrix} = (\mathbf{X}^\intercal \mathbf{X} + \alpha I')^{-1} \mathbf{X}^\intercal \mathbf{y}. \tag{4}$$

I' is the identity matrix, where the most right, most bottom diagonal element has been set to 0, i.e.

$$I' = I - \begin{pmatrix} 0 & \dots & 0 \\ \vdots & \dots & \vdots \\ 0 & \dots & 1 \end{pmatrix}. \tag{5}$$

Using I' instead of I here bears the advantage that regularization is imposed on $\mathbf{w}_{out}$, but not on b_{out}. α is the corresponding regularization parameter controlling the extent to which the model is allowed to fit the training data precisely. It needs to be optimised individually for every training run (see also Section 2).

1.3 Training multi-ESN networks with linear regression

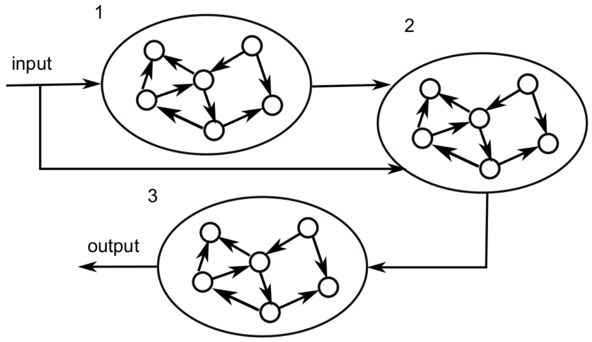

Figure 1: System Architecture of 3 connected Echo State Networks for which intermediate desired signals have been derived using backpropagation.

In order to train a multi-ESN architecture as shown in Figure 1 we need 3 ESNs, which we label as ESN 1 to ESN 3. We refer to the input signal, system matrix, output vector and target signal of ESN i as $v(n)^i$, W^i_{res}, $\hat{y}^i(n)$ and $y^i(n)$ respectively. When training multi-reservoir architectures using linear regression (assuming that we have suitable desired signals for all intermediate signals, be it analytical or trained) we proceed as follows. We first train ESN 1 using the original input vector $\mathbf{v}(n)$ as well as its assigned target signal $y^1(n)$ as shown in Section 1.2. We obtain the trained output of ESN 1 as

$$\hat{y}^1(n) = \mathbf{w}^1_{\text{out}} \mathbf{x}^1(n) + b^1_{\text{out}}. \tag{6}$$

For subsequent ESN, we feed the output of a trained ESN (as well as the original input in the case of ESN 2) as input to the next stage

$$v^{i+1}(n) = \hat{y}^i(n), \tag{7}$$

after which the training process is performed again using target signal $y^{i+1}(n)$. Using such an incremental approach, the resulting trained system is more robust to small errors made by ESNs closer to the input, opposed to an approach where all ESNs are trained using the desired signal of the previous reservoir stage as input.

1.4 Training with backpropagation

We train our proposed multi-reservoir architecture use stochastic gradient descent (SGD) with backpropagation to minimise the mean squared error e_{MSE} between the prediction of ESN 3 $\hat{y}^3(n)$ and the desired output signal $y(n)$.

$$e_{\text{MSE}} = \frac{1}{N}\sum_{n=1}^{N}(\hat{y}^3(n) - y(n))^2. \tag{8}$$

We do so by iteratively updating the readout vectors $\mathbf{w}_{out}^1$, $\mathbf{w}_{out}^2$ and $\mathbf{w}_{out}^3$ of ESN 1, 2 and 3 respectively, as we present minibatches of the input sequence $\mathbf{v}(n)$ to the networks.

We use the adaptive moment estimation (Adam) [2] learning rule, an extension of stochastic gradient descent (SGD) as follows. For every minibatch presented to the network, we update the output weight vector $\mathbf{w_{out}^i}$ of ESN i as

$$\mathbf{w}_{out}^i = (1-\lambda)\mathbf{w}_{out}^i - \eta\frac{\hat{m}_n}{(\sqrt{\hat{v}_n} + 10^{-8})}. \tag{9}$$

The hyperparameter η is used to control the stepsise towards the estimated minimum and is called *learning rate.* λ is another hyperparameter that regularises the learning process and is referred to as the *weight decay.* $\hat{\mathbf{m}}_n$ and $\hat{\mathbf{v}}_n$ are the bias-corrected first and second moment gradient estimates

$$\hat{\mathbf{m}}_n = \frac{\mathbf{m}_n}{1-(\beta_1)^n}, \tag{10}$$

where

$$\mathbf{m}_n = \beta_1\mathbf{m}_{n-1} + (1-\beta_1)\overline{\mathbf{g}^i} \tag{11}$$

and

$$\hat{\mathbf{v}}_n = \frac{\mathbf{v}_n}{1-(\beta_2)^n} \tag{12}$$

where

$$v_n = \beta_2\mathbf{v}_{n-1} + (1-\beta_2)(\overline{\mathbf{g}^i}\circ\overline{\mathbf{g}^i})1-\beta_2^n. \tag{13}$$

$\circ$ denotes the Hadamard product, the normalised gradient vector $\overline{\mathbf{g}^i}$ is computed as

$$\overline{\mathbf{g}^i} = \frac{\mathbf{g}^i}{||\mathbf{g}^i||_2} \tag{14}$$

where $||\,||_2$ is the 2-norm and

$$\mathbf{g}^i = \nabla_{\mathbf{w}^i} e_{\text{mse}} = \begin{pmatrix} \frac{\partial e_{\text{mse}}}{\partial w_1^i} \\ \frac{\partial e_{\text{mse}}}{\partial w_2^i} \\ \vdots \end{pmatrix} \tag{15}$$

is the gradient matrix with e_{mse} between $\hat{y}^3(n)$ and $y(n)$ with respect to the weight vector $\mathbf{w}^i_{out}$, as found by backpropagation through time (BPTT) [3]. As we implement our experiments in Pytorch [4], the step of gradient computation is performed automatically for us by the framework. β_1 and β_2 are hyperparameters controlling how strong the current gradient is incorporated into the actual first and second order moment gradient estimates. For more details on Adam, refer to [2], for chosen hyperparameter values, refer to Section 2.

To additionally improve convergence while training, we use an approach inspired by batch normalisation [5] during training, where we shift and scale the input for each ESN. If we denote the input to ESN i as $v^i(n)$ then we compute the normalised ESN input $v^i_{\text{norm}}(n)$ to be fed to ESN i as

$$v^i_{\text{norm}}(n) = \gamma^i \cdot v^i(n) + \delta^i, \tag{16}$$

where γ^i and δ^i are parameters to be optimised by means of gradient updates as shown in Equation 9, using gradients

$$\frac{\partial e_{\text{mse}}}{\partial \gamma^i}$$

and

$$\frac{\partial e_{\text{mse}}}{\partial \delta^i}.$$

respectively. Therefore, this setup learns a suitable normalization for intermediate reservoir signals. When transferring our training approach to physical reservoirs, one could work with modulators and adders to adjust the input signal for each reservoir correspondingly.

1.5 Deriving targets for linear regression

After having trained a multi-ESN architecture using backpropagation, we extract the full output sequences for the intermediate outputs of ESN 1 and ESN 2 on which we in the can be set as desired signals d_1 and d_2 for a new set of ESNs with different random weights, such that

$$y^1 = \hat{y}^1_{\text{bprop}}, \tag{17}$$

and

$$y^2 = \hat{y}^2_{\text{bprop}}, \tag{18}$$

where $\hat{y}^i_{\text{bprop}}$ denotes the output of ESN i trained by means of backpropagation as shown in Section 1.4. That way, using our derived target signals, we can train our given multi-reservoir architecture as shown in Section 1.3.

2 Used hyperparameters

2.1 ESN

For both the monolithic ESN as well as the multi-reservoir architecture with analytical targets, the validation set was used to find the hyperparameters that are intrinsic to the reservoir: spectral radius, input scaling, and bias scaling, leak rate, as well as the sparsity of the reservoir input and connection matrices. To tune the hyperparameters of our Echo State Networks, we have used the python package hyperopt [6], which attempts to find good hyperparameter configurations for computational models through the usage of surrogate modeling. Essentially, hyperopt samples from the hyperparameter space of the models error function, by computing the model errors of preselected points, i.e. hyperparameter sets. Based on these obtained errors it attempts to approximate the model error function by means of a surrogate model. This surrogate model of the model error surface can then be used to find a good set of hyperparameters. In order to account for varying reservoir weights, when sampling a single point in hyperparameter space, we supply the surrogate model with the average model error over 5 different random sets of reservoir weights. In general, the more points are sampled from the hyperparameter space, the more accurate the approximation, and the better the found set of hyperparameters. For our multi-ESN network, we sample 10000 times from the hyperparameter space for each of our 100-node ESNs. For the 300 node monolithic baseline ESN, we use the same amount of overall sampling operations and sample 30000 times to find a good set of hyperparameters. Table 1 shows the finally used hyperparameters for all ESN types.

	spec. rad.	in. scal.	bias scal.	leak r.	in. sprs.	conn. sprs.	nonl.
mono. ESN	1.1	0.9	1.0	1.0	0.65	0.89	tanh
ESN 1	0.9	0.5	0.6	1.0	0.51	0.95	ELU
ESN 2	0.7	0.9	0.8	1.0	0.96	0.86	ELU
ESN 3	1.1	1.0	0.1	0.8	0.92	0.68	ELU

Table 1: Used hyperparameters for all ESN types. Nonlinearities for ESN 1, 2 and 3 have been set as ELU to ensure sufficient gradient flow when using backpropagation. All remaining hyperparameters have been found using hyperopt [6].

2.2 Linear regression

The regularisation strength α was tuned individually for each reservoir using 5-fold crossvalidation on the train set. Possible values for α were evaluated on a logarithmic scale between 10^2 and 10^{-13}.

2.3 SGD with Backpropagation

We have tuned the minibatch sise, the training time in epochs, the learning rate η, as well as the weight decay λ. For the moment decay rates β_1 and β_2, Pytorch default values have been used. We have initialised all readout weights using orthogonal initialization [7]. We found that, despite the Adam learning rule is used, our results improved when dividing η by 2 after half of the total epochs trained. Furthermore we have found that the best results are achieved when not shuffling minibatches, but rather presenting them in the order dictated by the input sequence. This makes sense since presenting minibatches in their intended order allows the network to utilise its memory of input beyond the current minibatch. We applied a simple form of early stopping: Every 10 epochs in the training process, we recorded a snapshot of the current weights used in the network as well as the validation score it obtained. After 120 epochs, we chose the weight set with the lowest validation score. Finally, while we have experimented with the addition of artificial gradient noise [8], we found that the best results were achieved without adding any additional noise. Table 2 lists all found and set parameters for our backpropagation approach.

batch size	training epochs	η	λ	shuffle	init	β_1	β_2
60	120	$5/2.5 \cdot 10^{-4}$	10^{-4}	False	ortho.	0.9	0.999

Table 2: Used hyperparameters for training our proposed multi-ESN architecture using backpropagation

References

[1] Arthur Hoerl and Robert Kennard. Ridge regression: Biased estimation for nonorthogonal problems. *Technometrics*, 12(1):55–67, 1970.

[2] Diederik P Kingma and Jimmy Ba. Adam: A method for stochastic optimization. In *Proceedings of the International Conference on Learning Representations (ICLR)*, 2014.

[3] Paul Werbos. Backpropagation through time: what it does and how to do it. *Proceedings of the IEEE*, 78(10):1550–1560, 1990.

[4] Adam Paszke, Sam Gross, Francisco Massa, Adam Lerer, James Bradbury, Gregory Chanan, Trevor Killeen, Zeming Lin, Natalia Gimelshein, Luca Antiga, et al. Py-

torch: An imperative style, high-performance deep learning library. In *Advances in Neural Information Processing Systems*, pages 8024–8035, 2019.

[5] Sergey Ioffe and Christian Szegedy. Batch normalization: Accelerating deep network training by reducing internal covariate shift. In *Proceedings of the International Conference on Learning Representations (ICLR)*, 2015.

[6] James Bergstra, Brent Komer, Chris Eliasmith, Dan Yamins, and David D Cox. Hyperopt: a python library for model selection and hyperparameter optimization. *Computational Science & Discovery*, 8(1):014008, 2015.

[7] Andrew M Saxe, James L McClelland, and Surya Ganguli. Exact solutions to the nonlinear dynamics of learning in deep linear neural networks. In *Proceedings of the International Conference on Learning Representations (ICLR)*, 2013.

[8] Arvind Neelakantan, Luke Vilnis, Quoc V Le, Ilya Sutskever, Lukasz Kaiser, Karol Kurach, and James Martens. Adding gradient noise improves learning for very deep networks. In *Proceedings of the International Conference on Learning Representations (ICLR)*, 2015.
